# Oncogenic Potential of Epstein‐Barr Virus in NK and NKT Cells Contribute to the Rapid Deterioration of Hemophagocytic Lymphohistiocytosis

**DOI:** 10.1002/jmv.70481

**Published:** 2025-07-02

**Authors:** Tingting Cui, Mingzhu Huang, Yuan Wang, Zhengfang Lin, Xiaoling Su, Weidong Li, Qi Luo, Kaiyi Li, Chunyan Wang, Runhui Zheng, Zhongfang Wang

**Affiliations:** ^1^ State Key Laboratory of Respiratory Disease & National Clinical Research Center for Respiratory Disease, Guangzhou Institute of Respiratory Health the First Affiliated Hospital of Guangzhou Medical University, Guangzhou Medical University Guangzhou China; ^2^ Guangzhou National Laboratory, Guangzhou International Bio Island Guangzhou China; ^3^ Guangdong Provincial Second Hospital of Traditional Chinese Medicine Guangzhou China; ^4^ Key Laboratory of Emergency and Trauma of Ministry of Education, Engineering Research Center for Hainan Biological Sample Resources of Major Diseases, Key Laboratory of Tropical Cardiovascular Diseases Research of Hainan Province The First Affiliated Hospital of Hainan Medical University Hainan China; ^5^ Department of Clinical Laboratory Dongguan Maternal and Child Health Care Hospital Dongguan China; ^6^ Department of Hematology the First Affiliated Hospital of Guangzhou Medical University Guangzhou China; ^7^ Department of Hematology the Fifth Affiliated Hospital of Guangzhou Medical University Guangzhou China

**Keywords:** EBV oncogenicity, EBV‐HLH, EBV‐infected NK/NKT, host immunity

## Abstract

The rapid deterioration and fatal outcomes in Epstein‐Barr virus (EBV)‐related hemophagocytic lymphohistiocytosis (HLH) remain poorly understood. This study aimed to elucidate the key factors contributing to the progression of EBV‐HLH by comparing EBV cellular tropism and host immune responses between survivors and deceased patients. Compared to healthy individuals, acute HLH patients exhibited impaired natural killer (NK) cell activity, which improved during the recovery phase. However, deceased patients demonstrated heightened NK cell activity and increased EBV loads during the deterioration phase. Additionally, deceased patients had elevated EBV‐specific T cell responses without cytokine storm, suggesting other factors beyond host immunity contribute to HLH deterioration. Notably, in deceased cases, EBV infection spread to NK and NKT cells with a highly proliferative profile, whereas it was limited to B cells in survivors. Furthermore, EBV‐infected NK and NKT cells displayed a higher percentage of copy number variations and significant enrichment in canonical cancer pathways compared to noninfected cells, indicating their oncogenic potential and possible contribution to HLH deterioration. These findings provide insights into the pathogenesis of EBV‐HLH and may guide the development of targeted therapeutic strategies.

## 1 Introduction

Epstein‐Barr virus (EBV)‐associated HLH (EBV‐HLH), in particular, is linked to a grave prognosis, often due to persistent or relapsing EBV infections [1]. Studies have underscored the significantly lower 5‐year survival rate for EBV‐HLH patients at 25.1%, contrasting sharply with the rates for those with HLH secondary to autoimmune diseases (82.4%) or other infections (78.7%) [2]. The distinction between EBV‐infected and noninfected HLH has been further highlighted by You et al., who identified non‐EBV infection‐associated HLH as a subgroup with a more favorable prognosis, where 70% of patients achieved complete recovery without relapse [3]. EBV mainly infects human B cells, establishing a silent lifelong infection. In contrast, infection of T and natural killer (NK) cells by EBV invariably results in life‐threatening diseases. Chronic active EBV (CAEBV) disease with EBV infection in T and NK cells is associated with a more aggressive clinical course and higher mortality compared to B‐cell related CAEBV [4]. Notably, NK cell CAEBV is associated with a higher risk of progression to aggressive NK cell leukemia or extranodal NK/T lymphoma [4, 5].

EBV‐HLH progresses and relapses rapidly, leading to high mortality rates. It is imperative to compile and analyze the clinical readouts to improve the identification the progress of EBV‐HLH. A nomogram based on ferritin, CD3‐CD16 + CD56 + %, anti‐EBV‐nuclear antigen (NA)‐IgG, Interleukin‐6 (IL‐6) and Interleukin‐6 (IL‐10) has been reported to be a robust predictive detective parameter for EBV‐HLH [6]. Yao et al. identified an improved HLH index, containing higher soluble interleukin‐2 receptor alpha subunit (sCD25), estimated glomerular filtration rate and lower procalcitonin, as indicative of a poor prognosis [7]. Recently, a nomogram combining the maximal standardized uptake value (SUVmax) ratio, copies of plasma EBV‐DNA, and IFN‐γ has demonstrated predictive power for mortality in EBV‐HLH patients [8]. Despite these advancements, the mechanisms underlying the susceptibility of EBV‐HLH to relapse and the factors contributing to its rapid clinical deterioration remain elusive.

EBV infection is controlled by antiviral immune responses, particularly by virus‐specific T cells and innate NK cell cytotoxicity. Impaired immune surveillance due to dysregulated T and NK cell cytotoxic of EBV infection results in EBV‐related diseases, such as EBV‐HLH, lymphoproliferative disorders (LPDs) and CAEBV [9]. However, recent evidence indicates that CAEBV‐infected patients exhibit high levels of T/NK cell activation without inhibition of CD4 + T cell functional differentiation [10]. Carvelli et al. have also noted an activated NK cell phenotype with increased expression of CD69, ICAM‐1, HLA‐DR, and CCR5 in EBV‐HLH patients, yet with cytotoxicity comparable to healthy controls [11]. Thus, the key factors contributing to the poor outcomes in EBV‐HLH require further investigation.

To identify the contributors to the prognosis of EBV‐HLH, we compared the cell tropism of EBV infection and the host immune response in EBV‐HLH patients who recovered versus those who did not. We first investigated NK cell activity in healthy donor and EBV‐HLH patients. We then detected the EBV‐specific T cell responses, and cytokine levels at different stage of EBV‐HLH progression to confirm the role of immune cell response in controlling EBV infection. Furthermore, utilizing single‐cell RNA transcriptomics, we analyzed the immune profile landscape between surviving and deceased patients, which helped to identify the key factors for the deterioration of EBV‐HLH. This study provides a novel insight into the progression of EBV‐HLH, which is of great value for future therapeutic strategies for EBV‐HLH.

## 2 Patients and Methods

### 2.1 Patient Cohort

According to HLH‐2004 criteria (fever, bicytopenia, hyperferritinemia, hemophagocytosis, splenomegaly, elevated sCD25, hypofibrinogenemia, and hypertriglyceridemia) with active EBV infection, 22 patients from the First Affiliated Hospital of Guangzhou Medical University were diagnosed as EBV‐HLH and recruited in this study. Among them 16 patients were categorized into acute phase (*n* = 16), 9 into remission phase (*n* = 9, including 4 responders and 5 re‐admitted cases), and 3 into deterioration phase (*n* = 3, including 2 deterioration cases and 1 re‐admitted case, Table 1). The median age of these 22 patients was 34 years (interquartile range (IQR), 22–46 years) and 72.7% (16/22) were male. Meanwhile, to confirm the degranulation level of NK cells in healthy individuals, 57 healthy individuals were recruited and used as control. The healthy group with a median age of 30 years (interquartile range (IQR), 28–31 years) and 50.9% (29/57) were male. The blood samples for all the EBV‐HLH patients and healthy individuals were collected. This study was approved and monitored by the GMUH Ethics Committee (No. 2021‐78).

**Table 1:** Clinical and laboratory diagnosis for HLH at various stages of disease progression.

Phase	Cases	Fever°C	Bicytopenia	TG mmol/L	Ferritin ng/mL	Fibrinogen g/L	sCD25 U/ml	Hemopha‐gocytosis	Spleno‐megaly
Acute phase	P1	38.5	+	4.29	2174	1.48	1929	ND	+
	P2	39	+	2.58	45159	1.6	162045	+	+
	P3	40	−	ND	8110	1.76	ND	+	+
	P4	38.7	+	4.78	1812	1.42	−	+	+
	P5	39	+	5.77	22882	2.31	12025	+	+
	P6	38.2	−	5.93	> 2000	1.2	≥ 2400	+	ND
	P7	40.7	+	4.47	14147	1.15	23726	ND	+
	P8	38.4	+	4.1	> 2000	0.74	ND	ND	+
	P9	41.2	+	1.22	> 2000	3.48	ND	+	+
	P10	38.7	+	ND	25626	2.43	2500	+	−
	P11	38.1	+	3.7	> 500	ND	ND	ND	+
	P12	36.2	+	2.31	1650	2.9	6534	+	+
	P13	39	+	4.01	ND	0.81	ND	+	+
	P14	39.7	+	3.7	ND	1.97	ND	+	ND
	P15	38.8	−	ND	2726.39	4.17	135591	+	+
	P16	38.4	+	1.82	ND	3.66	53200	+	+
Recovery phase	P1	36.8	+	1.87	1751.43	2.27	1500	ND	+
	P2	36.2	−	4.57	1300.07	2.2	59	−	−
	P3	36.2	−	4.41	76.98	2.93	−	ND	ND
	P16	−	−	3.32	ND	1.99	ND	ND	+
	P17	−	−	1.72	ND	5.75	1018	−	−
	P18	−	−	ND	824.4	5.36	ND	ND	+
	P19	−	−	0.81	70.49	1.59	ND	+	+
	P20	−	−	1.41	859.8	4.95	−	−	+
	P21	39.4	−	2.21	886.4	4.57	ND	−	−
Deterioration phase	P1	39.1	+	3.68	8478.82	0.89	8364	ND	+
	P2	39.4	+	ND	> 40000	1.18	4409	ND	ND
	P22	39.5	+	ND	ND	1.33	ND	+	+

Abbreviations: −, negative; +, positive; ND, not tested; P, patient.

### 2.2 NK Cell Degranulation Assay

The NK cell degranulation was determined by the cell surface expression of CD107a as described [12]. Fresh peripheral blood mononuclear cells (PBMCs) were incubated with K562 cell at a ratio of 1:1 for 3 h at 37°C and 5% CO_2_. After incubation, the cells were stained with LIVE/DEAD^TM^ Fixable Aqua dead cell sting kit for 15 min (ThermoFisher scientific, Cat# L34957). Then surface‐staining was performed for 30 min with the following antibodies: FITC anti‐human CD3 (BioLegend, clone UCHT1, 1:200, Cat# 300406), PE ‐ Cy7 anti‐human CD56 (BD Biosciences, clone B159, 1:200, Cat# 557747), APC anti‐human CD107a (BD Biosciences, clone H4A3, 1:200, Cat# 560664). Thereafter, cells were resuspended and acquired using a FACSAria III instrument (BD Biosciences) and analyzed with FlowJo, version 10.0 (Treestar).

### 2.3 EBV‐Specific T Cell Responses of Patients With EBV‐HLH

To elicit virus‐specific T cell responses, PBMCs were stimulated with EBV peptide pools containing 1623 15‐mer peptides of EBV proteins at 250 nM per peptide in the presence of 10‐U/mL rIL‐2 (Sigma, St Louis, MO, USA) and 1‐µM GolgiPlug (BD Biosciences, San Diego, CA, USA) overnight at 37°C and 5% CO_2_. An intracellular cytokine staining assay was then performed. Briefly, the cells were harvested and incubated with LIVE/DEADTM Fixable Aqua dead cell sting kit for 15 min (ThermoFisher scientific, L34957). The cells were then surface‐stained with the following antibodies: FITC anti‐human CD3 (BioLegend, clone UCHT1, 1:200, Cat# 300406), APC ‐ H7 anti‐human CD4 (BD Biosciences, clone RPA‐T4, 1:200, Cat# 560158), and PerCP ‐ Cy5.5 anti‐human CD8 (BD Biosciences, clone RPA‐T8, 1:200, Cat# 560662). Following fixation/permeabilization (BD Biosciences), intracellular staining was performed with PE ‐Cy7 anti‐human TNF‐α (BD, clone MAb11, 1:200, Cat# 557647) and APC anti‐human IFN‐γ (BD Biosciences, clone B27, 1:200, Cat# 554702). Finally, cells were acquired using a FACSAria III instrument (BD Biosciences) and analyzed with FlowJo software (Treestar).

### 2.4 Measurement of Cytokines in Plasma

Cytokines (i.e., interleukin [IL]‐2, IL‐4, IL‐6, IL‐8, IL‐12, macrophage inflammatory protein‐1α [MIP‐1α], IFN‐γ, and TNF‐α) for plasma samples were measured using a cytometric bead array Kit (CBA, BD Biosciences), following the manufacturer's instructions. Eight types of diluted cytokine capture beads (1:50) were freshly mixed in equal amounts (50‐µL bead per sample). Then, 50 µL of the mixed beads was incubated with 50 µL of standards or samples at room temperature for 1 h, followed by incubation with 50 µL of phycoerythrin (PE) detection reagent for 2 h. Thereafter, the beads were analyzed on a FACSVerse flow cytometer (BD Biosciences).

### 2.5 Single‐Cell RNA Sequencing Analysis

Three patients were selected for Single‐cell RNA sequencing (scRNA‐seq) to represent divergent clinical outcomes. Two deceased patients with deterioration phase and one survivor with complete recovery. To discover which viral or host factors deteriorate HLH progression, samples collected at three timepoints (T1: acute phase, T2: remission phase, T3: deterioration/recovery phase) for each patient were sent for scRNA‐seq. This design enabled comparative analysis of EBV tropism, immune cell activation, and oncogenic pathways between fatal and nonfatal cases, with longitudinal sampling capturing disease progression dynamics. scRNA‐seq libraries were generated following the recommended protocol for 10× Genomics' scRNA‐seq platform and using Chromium Next GEM Single‐Cell 3′ Reagent Kit (version 3.1), and sequencing data were collected using standard Illumuna. Cellranger (version 3.1.0) and were aligned to the human genome GRCh38. Seurat was used on Cell Ranger outputs, and cell filtering, clustering, and visualization were performed with it. Cell type was annotated using marker genes that were collected from a previous study [13]. The scores for 10 cancer hallmark pathways were calculated using the “Gene Set Variation Analysis (GSVA)” package with the “single‐sample gene set enrichment analysis (ssgsea)” method. The copy number variation was performed using the “Copykat” package with the default settings.

### 2.6 FlowRNA for Detecting EBV‐Encoded Small RNAs (EBERs)

To determine EBV infection in the lymphocyte subgroup, flowRNA assay was performed following the manufacturer's instruction. Briefly, 1 × 10^6^ PBMCs were firstly incubated with LIVE/DEAD and surface stained with PE anti‐human CD3 (BD Biosciences, clone UCHT1, 1:200, Cat# 555333), BV421 anti‐human CD4 (Biolegend, clone OKT4, 1:200, Cat# 317434), and PE ‐ Cy7 anti‐human CD56 (BD Biosciences, clone B159, 1:200, Cat# 557747). After fixation/permeabization, the target RNA was hybridized using target probe sets (custom designed by PrimeFlow RNA, Thermo‐Fisher Scientific) for 2 h. Thereafter, the cells were sequential hybridized using the “PreAmplifier” and “Amplifer”, followed by hybridization with fluorescent label. Finally, the cells were acquired using a FACSAria III instrument (BD Biosciences) and analyzed using FlowJo (Treestar).

## 3 Results

### 3.1 Patient Characteristics

**Figure 1:**
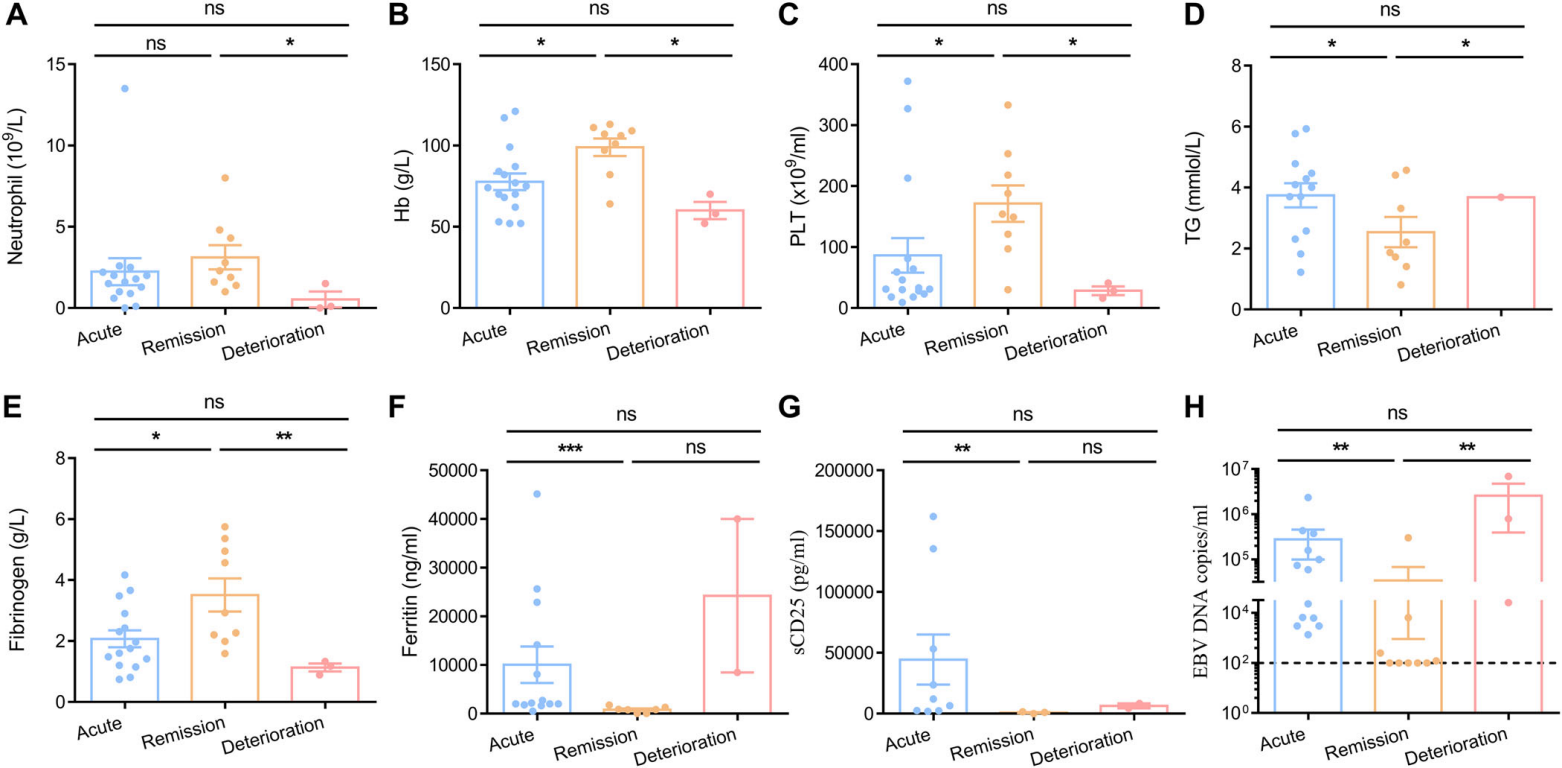
Deceased EBV‐HLH patients exhibited persistent EBV infection throughout the progression of the disease. Dynamics of neutrophil count (A), hemoglobin (Hb) concentration (B), platelet (PLT) count (C) in the peripheral blood of EBV‐HLH patients at different stage of disease progress (*n* = 16 in acute phase; *n* = 9 in remission phase; *n* = 3 in deterioration phase). Levels of triglycerides (TG) concentration (D), fibrinogen (E), ferritin (F) and sCD25 (G) in the serum of EBV‐HLH patients. (H) Plasma EBV‐DNA copy numbers in all patients. The dotted line represented the clinically determined threshold for a positive diagnosis of EBV infection in plasma samples.

As shown in Table 1, there were 16 cases that met the HLH‐2004 criteria and were classified as acute HLH. After treatment, 9 patients (four patients derived from acute phase, 5 re‐admitted cases) achieved remission with resolution of fever, increased leukocyte/platelet counts, reduced ferritin/sCD25 levels (Figure 1), indicating the remission phase. Following continuous monitor, two patients (Patient 1, P1 and Patient 2, P2) from remission phase went through a rapid relapse with life‐threatening features, including persistent high fever, recurrence of cytopenia, high ferritin, and rising EBV‐DNA (Figure 1). Another patient experienced the same features and succumbed to the disease, thus, they were categorized into the deterioration phase. In comparison to patients in the acute HLH phase (*n* = 16), those in the remission phase (*n* = 9) displayed normal body temperature, increased leukocyte counts, and decreased levels of sCD25 and ferritin. However, patients in the deterioration phase (*n* = 3) after relapse exhibited a higher body temperature (> 39°C), lower levels of leukocytes, hemoglobin, and platelet count, and a significant increase in ferritin and sCD25 (Table 1, Figure 1A–G), indicating an unfavorable outcome of HLH in the deteriorating phase.

### 3.2 Deceased EBV‐HLH Patients Exhibited Elevated EBV Loads at Deterioration Phase

To investigate EBV infection during different stages of EBV‐HLH, we measured EBV viral loads. The results indicated that patients in the acute phase exhibited a high level of EBV infection (2.8 × 10^5^ copies/mL). During the remission phase, most patients showed clearance of EBV infection. However, the two deceased patients (P1 and P2) maintained a high level of EBV load during the remission phase, which further increased to an even higher level during the deterioration phase (Figure 1H). This suggests that the persistence of EBV infection may be a major contributing factor to the fatal outcome of HLH.

### 3.3 NK Dysfunction, Lower EBV‐Specific T Cell Levels, and Hypercytokinemia Were Not Observed at the Deterioration Timepoint

**Figure 2:**
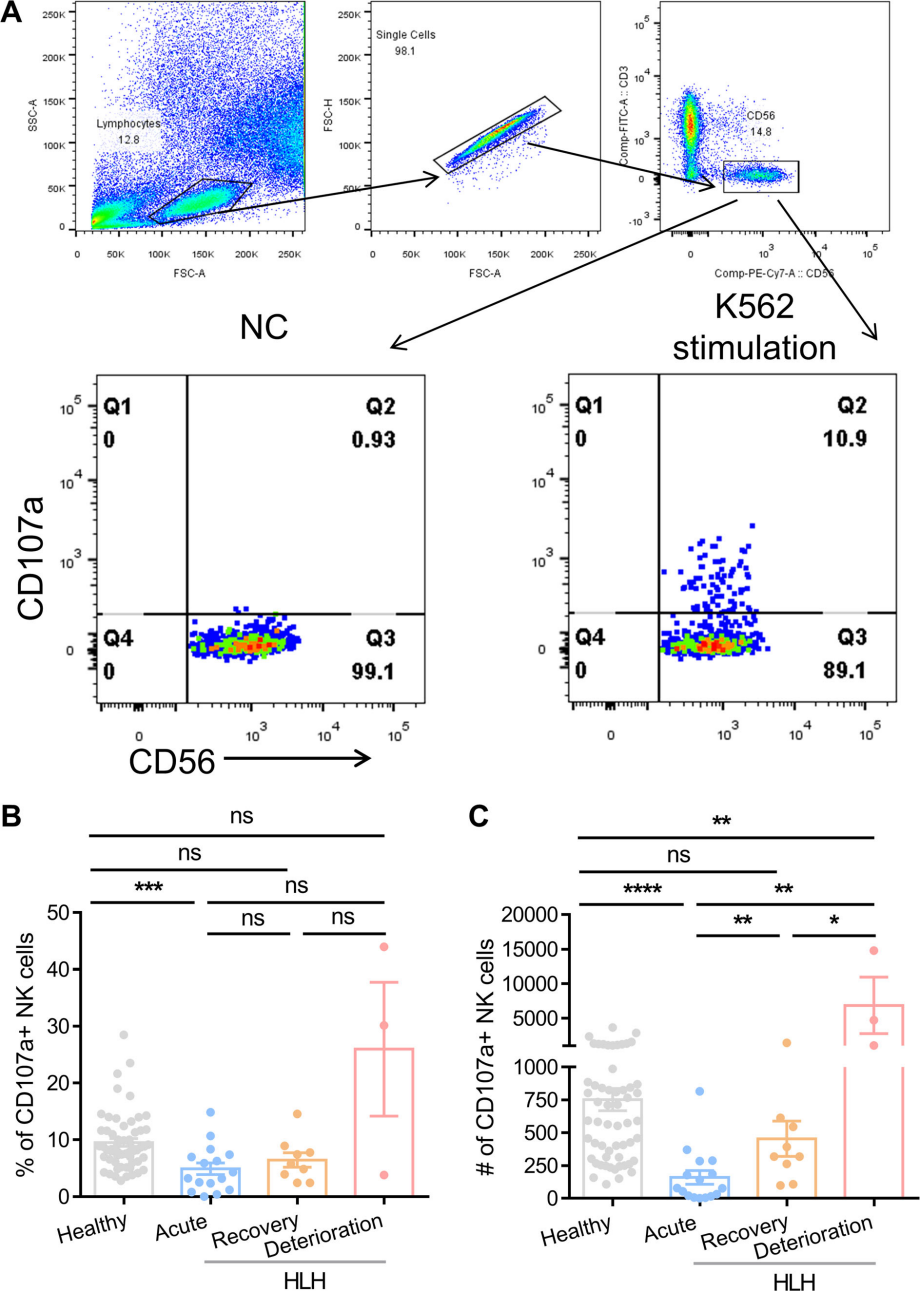
Deceased patients in the deterioration phase exhibited heightened levels of NK cell activity. (A) Gating strategy for NK cell degranulation assay with K562 cells. The ability of NK cell degranulation for the healthy individuals (*n* = 57) and EBV‐HLH patients (*n* = 16) at different stages of disease onset was presented in percentages (B) and absolute cell numbers 4 x 10^5^ PBMCs (C).

To examine the impact of NK cell activity on the progression of EBV‐HLH, we initially compared the NK cell degranulation capacity, as measured by the number of CD107a+ NK cells per 4^10^5^ PBMCs, between healthy individuals and acute HLH patients (Figure 2A). The findings revealed that acute HLH patients (159 CD107a+ NK cells per 4^10^5^ PBMCs, *n* = 16) had significantly lower degranulation capacity compared to healthy individuals (752 CD107a+ NK cells per 4^10^5^ PBMCs, *n* = 57). Following treatment, NK cell activity showed a significant increase during the remission phase (454 CD107a+ NK cells per 4^10^5^ PBMCs). Surprisingly, deceased patients in the deterioration phase exhibited heightened levels of NK cell degranulation (6850 CD107a+ NK cells per 4^10^5^ PBMCs), surpassing even that of healthy individuals (Figure 2B,C). Deceased patients also had high level of NK cytotoxicity at deterioration phase (Supporting Information S1: Figure S1).

**Figure 3:**
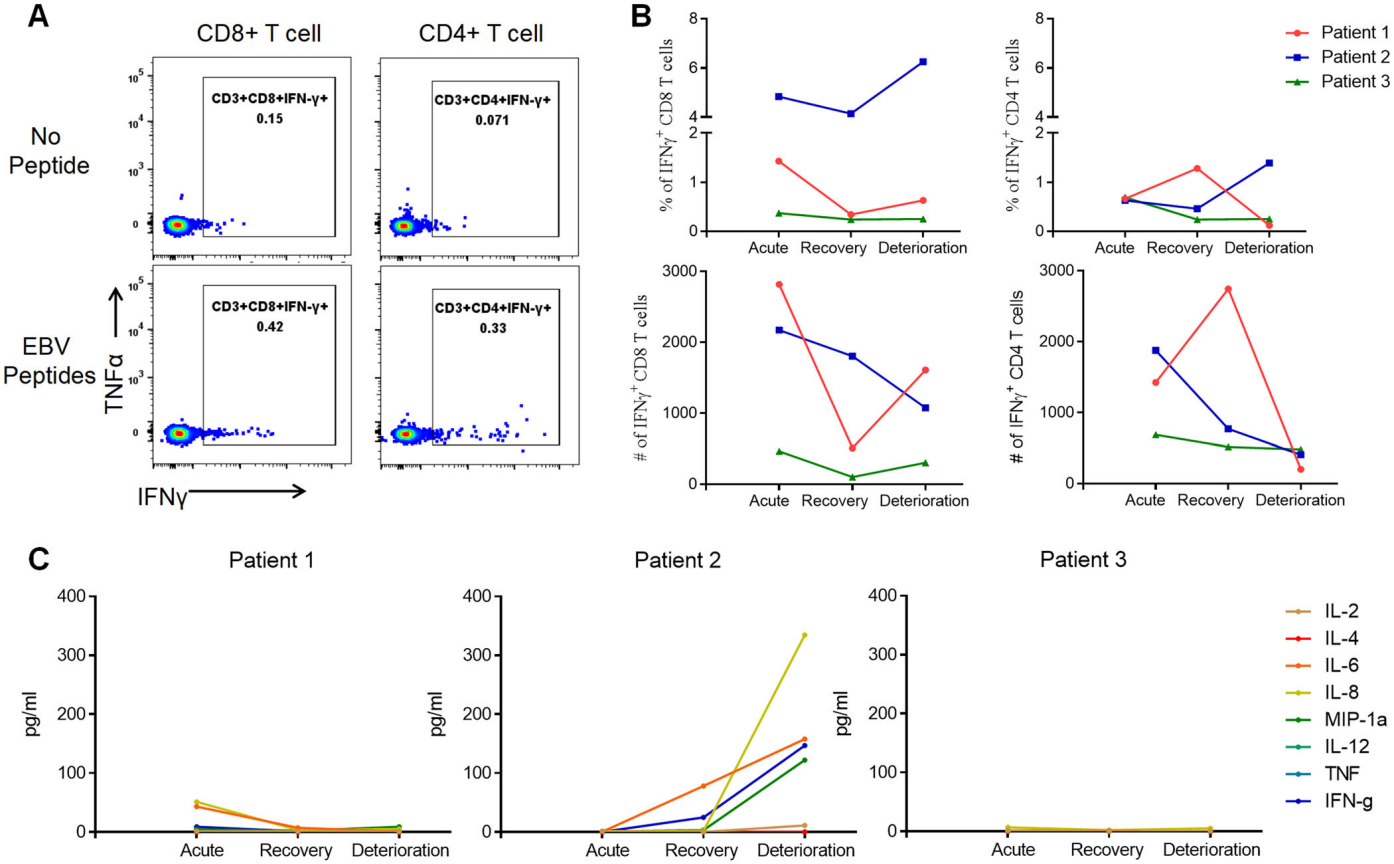
Functionality of T cells, cytokinemia measurement for the patients with three EBV‐HLH at the indicated timepoints. (A) Representative dot plots showing EBV‐specific T cells detected by IFN‐γ expression in CD4+ and CD8 + T cells after EBV peptide stimulation. (B) The percentages and number/million PBMCs of IFN‐γ + CD8 + T cells (*left panel*), and the percentages and number/million PBMCs of IFN‐γ + CD4 + T cells (*right panel*) of the patients. (C) Plasma concentration of IL‐2, IL‐4, IL ‐ 6, IL‐8, IL‐12, MIP‐1a, TNF‐α, and IFN‐γ of all patients at the indicated timepoints.

In addition, we compared the EBV‐specific T cell responses in two deceased patients (P1 and P2) and one fully recovered patient (Patient 3, P3) at different stages of the disease to investigate whether the deterioration of HLH is associated with a lack of T cell response, which plays a crucial role in controlling EBV infection. Since Patient 3 achieved full recovery without relapse, the fully recovery phase (referred to as timepoint 3, T3) was chosen as a control for the deterioration phase observed in P1 and P2. Meanwhile, we designated the acute phase as timepoint 1 (T1) and the remission phase as timepoint 2 (T2). We found that P1 and P2 had high EBV‐specific CD8 + T cell responses at T1 with 1.43% and 4.83% of IFN‐γ + CD8 + T cells, respectively, which maintained at a high level at T3 and higher than that for P3 (Figure 3A,B). Meanwhile, two deceased patients had higher CD4 + T cell responses at T3 than that for recovered patient (Figure 3B).

Furthermore, the results showed that only IL‐2, IL‐6, IL‐8, IFN‐γ, and MIP‐1α were elevated for P2 at T3, other cytokines containing IL‐4, IL‐12, TNF‐α were at low level for P2, as well as for P1 that all the cytokines kept at a low level at T3 (Figure 3C). The cytokines for P3 were all at low levels at T3, except for IL‐8 (5.16 pg/mL; Figure 3C).

Taken together, these data revealed that deceased patients in the deterioration phase exhibited a sustained high level of functional T and NK cell responses, which were insufficient to effectively control the EBV infection. Additionally, these patients had low levels of cytokines, suggesting that other factors contribute to the progression of disease deterioration.

### 3.4 Transcriptome Analysis Revealed That NK and NKT Subsets Increased in Percentage and Functionality at the HLH Deterioration Timepoint

**Figure 4:**
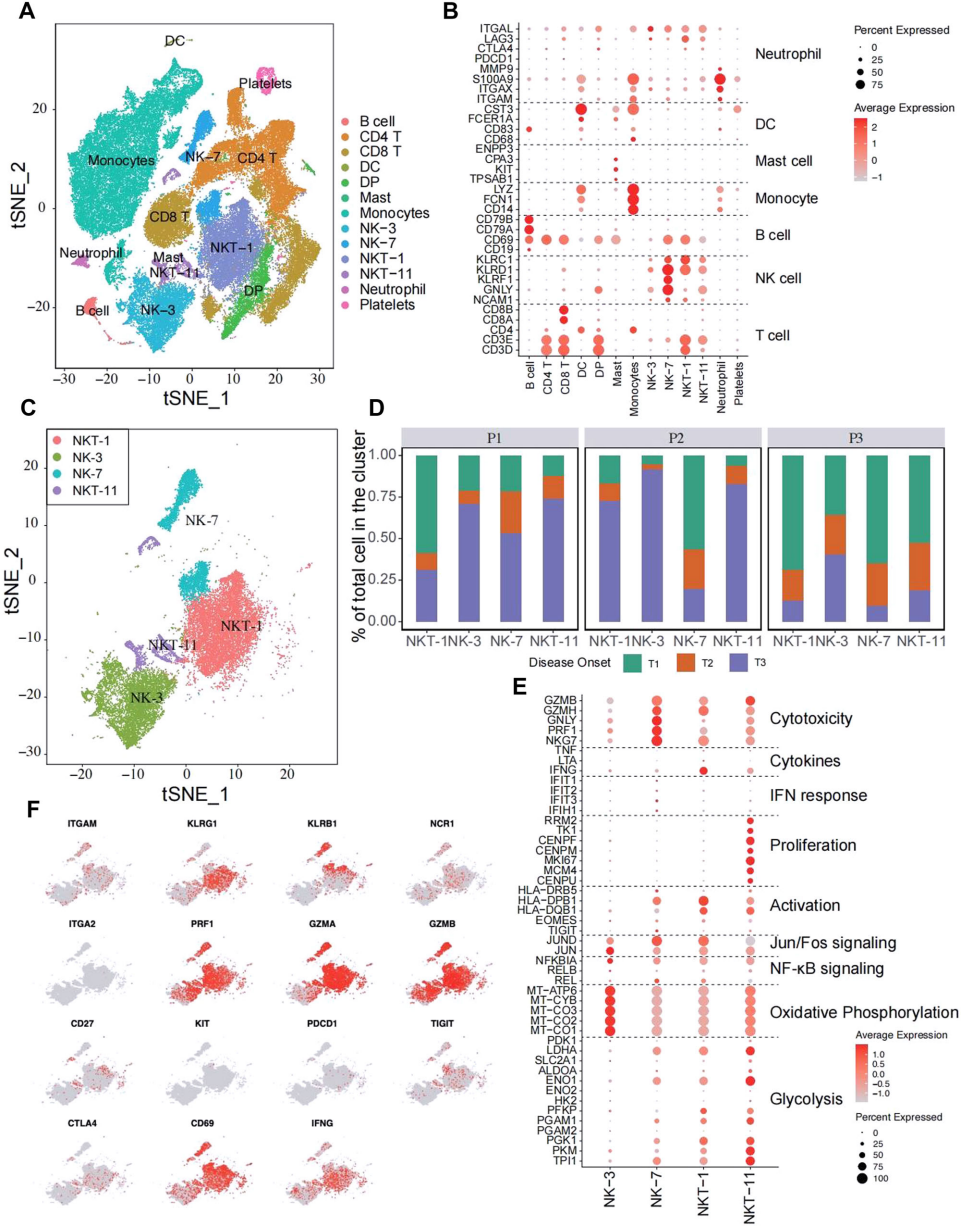
High‐throughput single‐cell RNA‐seq of the patients with HLH reveals the overgrowth of NK and NKT cells at the deterioration phase. (A) A *t*‐Distributed Stochastic Neighbor Embedding (tSNE) of 64 657 single cells defining 13 clusters. (B) The average expression (color scale) and the percentage of expressing cells (size scale) of marker genes in all clusters. (C) Reclustering of NK and NKT clusters. (D) Percentage of NK and NKT cells at different stages of disease onset among the three patients. (E) The average expression (color scale) and the percentage of expressing cells (size scale) of selected genes in the indicated clusters. (F) Expression of the selected genes across the reclustered tSNE plot, indicating the gene expression for maturation markers, activating, inhibitory receptors, and cytokine IFN‐γ and activation marker CD69.

To further discover which viral or host factors deteriorate HLH progression, scRNA‐seq for PBMCs of P1, P2, and P3 with different timepoints (T1‐T3) was performed. Thirteen clusters were defined (Figure 4A). Following the marker gene expression pattern in each cell cluster, four clusters presenting a high expression of the NK cell markers CD56, GNLY, KLRD1, and KLRC1, were defined. Among which, two clusters also simultaneously expressed T cell markers (i.e., CD3D and CD3E; Figure 4B). Therefore, these four clusters were designated as NK‐3, NK‐7, NKT‐1, and NKT‐11 (Figure 4B,C). Notably, the portion of each cluster within these four subsets differed greatly among the different timepoints (Figure 4D). The results showed that P1 and P2 had the highest proportions of NK‐3, NK‐7, NKT‐1, and NKT‐11 cells at T3. While, P3 showed the highest number of NK and NKT cells at T1 then continue reduced at T3. The NK‐7 and NK‐3 were highly featured by cytotoxic and oxidative phosphorylation signature, respectively (Figure 4E), NKT‐1 showed strong activation and a signature marked by the enrichment of Jun/Fos signaling, and NKT‐11 displayed a dual signature marked by the enrichment of proliferation and glycolysis genes (Figure 4E). Similarly, the expression of Prf1, GZMA, and GZMB, was highly presented in NK and NKT populations, indicating that they possessed a killing capacity. The activation marker CD69 was predominantly observed in NK‐7 and NKT‐1, whereas the transcripts of IFN‐γ were mainly observed in NKT‐1 (Figure 4F). Moreover, the expression of the checkpoint inhibitory receptors PD‐1, TIGIT, and CTLA4 were not obvious in NK and NKT cells. These results are consistent with the data shown in Figure 2 that deceased cases at T3 had a higher level of functionality of NK and T cells.

### 3.5 The Percentage of NK and NKT Cells With Lytic and Latent EBV Infection Substantially Increased at the HLH Deterioration Timepoint

**Figure 5:**
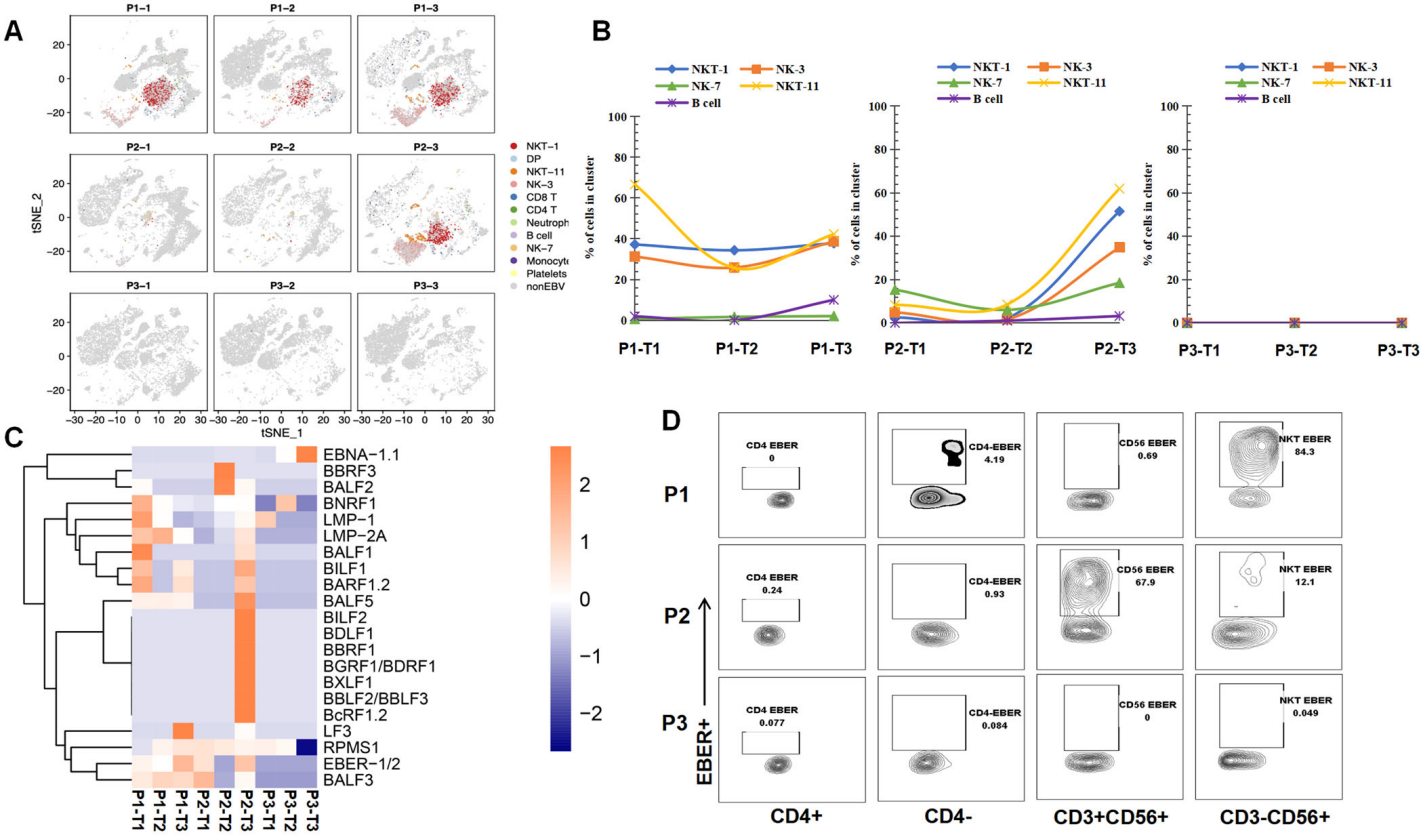
Identification of dynamic EBV cell tropism by scRNA‐seq and confirmation by flowRNA assay. (A) tSNE plots displaying the major cell clusters positive for EBV genes. (B) The percentage of EBV‐infected NK, NKT and B cell clusters is displayed for each patient at the indicated timepoints. (C) Different EBV genes detected by scRNA‐seq for the three patients at different stages of EBV‐HLH; the average expression of EBV genes is shown. (D) EBV EBER RNA detected by flowRNA assay for the indicated PBMC subsets at T3 of the three patients to confirm the cell tropism observed from scRNA‐seq data. Cells were multicolor‐stained for EBER and cell differentiation markers, including CD3, CD4, and CD56.

Importantly, high percentages of EBV RNA‐positive NKT‐1, NKT‐11, and NK‐3 were detected at T3 for P1 and P2 (Figure 5A). The infection in the NK and NKT clusters for P1 was persistent within the disease progression, which was 37%, 31% and 67% of NKT‐1, NK‐3 and NKT‐11, respectively, at T1 and 37%, 39% and 42% of NKT‐1, NK‐3 and NKT‐11, respectively, at T3 (Figure 5B, left panel). Additionally, the EBV infection of NK‐3, NKT‐1, and NKT‐11 for P2 was at low levels (< 10%) at T1 and T2, which sharply increased to 35%, 51%, and 62%, respectively, at T3 (Figure 5B, middle panel). No EBV RNA was observed for NK and NKT cells at all three timepoints for P3 (Figure 5B, right panel). In B cells, P1 showed low infection (1.89%) at T1, which rose to 10% at T3. P2 has no EBV infection in B cells at T1, but 3.19% of B cells were infected at T3 as the disease progressed. P3 exhibited no EBV RNA in B cells at all three timepoints (Figure 5B). Collectively, the highest proportion of NK‐3, NKT‐1, and NKT‐11 cells at T3 (Figure 4D), coupled with elevated infection rates in NK/NKT cells (but not B cells), strongly suggest that dynamic EBV expansion in NK/NKT subsets is closely associated with clinical deterioration.

Furthermore, *BLLF1*, *BALF3*, *BALF5*, *LF3*, *BARF1*, *EBER*, *EBNA‐1*, *LMP‐1*, *LMP‐2A*, and *RPMS1* were found to be the most frequently detected genes in the deterioration phase in P1 and P2 (Figure 5C), which resembled a canonical type II latency profile and the lytic cascade. Interestingly, no obvious difference was found between EBV‐infected and noninfected NK/NKT cells in terms of the function and expression of cytokines (Supporting Information S1: Figure S2), indicating that EBV infection did not influence the function of NK or NKT cells.

To confirm the EBV cell tropism observed by scRNA‐seq, a multicolor flow cytometry‐based assay (flowRNA) was established to simultaneously detect the abundantly expressed viral noncoding RNAs (EBER‐1/2) present in every infected cell for all patients at T3. The results showed that EBV‐infected 84.3% of CD3‐CD56 + NK cells, and 0.69% of CD3 + CD56 + NKT cells for P1 at T3 (Figure 5D). P2 infected 67.9% CD3 + CD56 + NKT cells, and 12.1% of CD3‐CD56 + NK cells. No EBER‐positive cells were observed in P3 (Figure 5D). These results are consistent with the results of the scRNA‐seq analysis.

### 3.6 EBV‐Infected Cells Presented Significant Enrichment in Canonical Cancer Pathways

**Figure 6:**
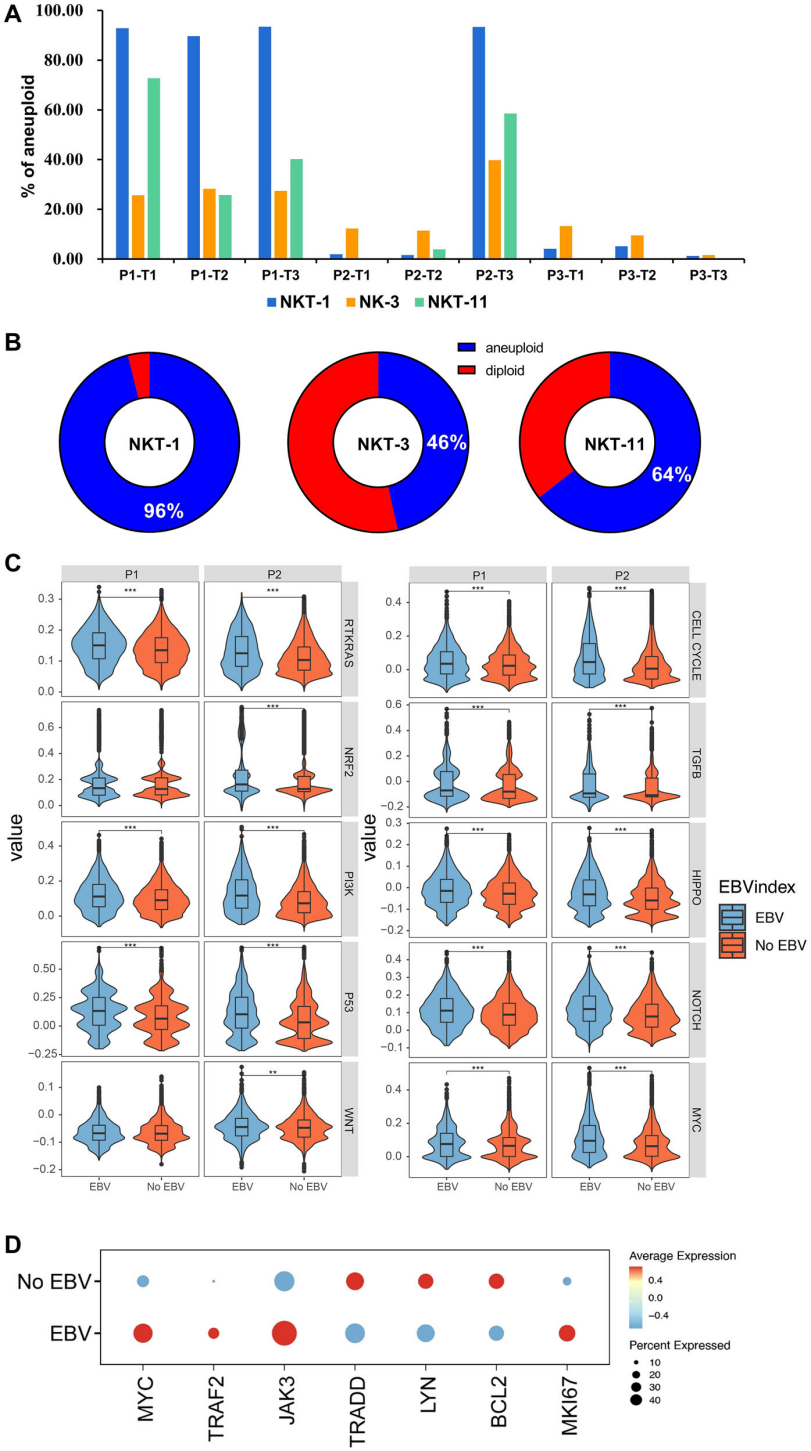
Single‐cell RNA‐seq identifies an oncogenesis of EBV‐infected NK and NKT cells. (A) CNVs of C1‐NKT, C3‐NK, and C11‐NKT clusters. (B) The percentage of aneuploidies for C1‐NKT, C3‐NK, and C11‐NKT clusters of the three patients at different stages of disease onset. (C) The percentage of aneuploidies for EBV‐infected C1‐NKT, C3‐NK, and C11‐NKT clusters. (D) Comparison of the enrichment of 10 canonical pathways, including cell cycle, Hippo signaling, Myc signaling, Notch signaling, oxidative stress response/Nrf2, PI‐3‐Kinase signaling, receptor‐tyrosine kinase (RTK)/RAS/MAP‐Kinase signaling, TGFβ signaling, p53, and β‐catenin/Wnt signaling between EBV‐infected and non‐EBV‐infected cells. (E) Expression of selected genes representing proto‐oncogenes, proliferation, and EBV viral carcinogenesis for EBV‐infected vs. non‐EBV clusters. **p < 0.01, ***p < 0.001.

Although functionality loss among EBV‐infected NK or NKT cells was not observed, the consequences of the infected NK and NKT cells were speculated given that EBV can transform B cells into lymphoma. Therefore, the transcriptional profiles of EBV + NK and NKT subsets were investigated. The results showed that highly infected NKT‐1, NK‐3, and NKT‐11 displayed quite high copy number variations (CNVs). At T3, the percentage of aneuploid cells for NKT‐1, NK‐3, and NKT‐11 was 90%, 23%, and 40%, respectively, for P1. For P2, the percentage of aneuploid cells for NKT‐1, NK‐3, and NKT‐11 was 91%, 40%, and 60% at T3, respectively (Figure 6A).

Furthermore, the correlation between EBV infection and CNVs was analyzed, and the data showed that 96% of EBV‐infected NK‐1 had CNVs, which was 46% and 64% for NK‐3 and NKT‐11, respectively (Figure 6B), indicating that EBV infection promoted the tumorigenesis of infected NK and NKT cells. The gene enrichment in 10 canonical cancer pathways was compared to confirm the tumorigenesis effect of EBV infection, and the results showed that EBV‐infected cells exhibited significantly higher enrichment in the canonical cancer pathways than that in non‐EBV‐infected cells (Figure 6C). The significant upregulation of the canonical cancer pathways was observed for infected NK and NKT cells (Supporting Information S1: Figure S3A).

These activated cells may proliferate as tumor cells because the CNV increased in infected NK and NKT subsets. By comparing differentially regulated genes between EBV‐infected and non‐EBV‐infected cells, genes associated with proto‐oncogenes transcription factor (*Myc*), proliferation (*MKI67*), and EBV LMP1‐related carcinogenesis (*TRAF2* and *Jak3)* were observed to be highly expressed in EBV‐positive cells. Conversely, EBV LMP1‐related (*TRAAD*) and LMP2A‐related (*Lyn*) carcinogenesis and antiapoptosis (*Bcl2*) genes were downregulated in EBV‐positive cells (Figure 6D). These results suggest that EBV infection in NK and NKT cells promote their carcinogenesis. The same results in gene expression could be observed at the disease progression level (Supporting Information S1: Figure S3B).

### 3.7 Pseudotime Analysis Traces the Possible Origin of NKT Cells to T Cells

**Figure 7:**
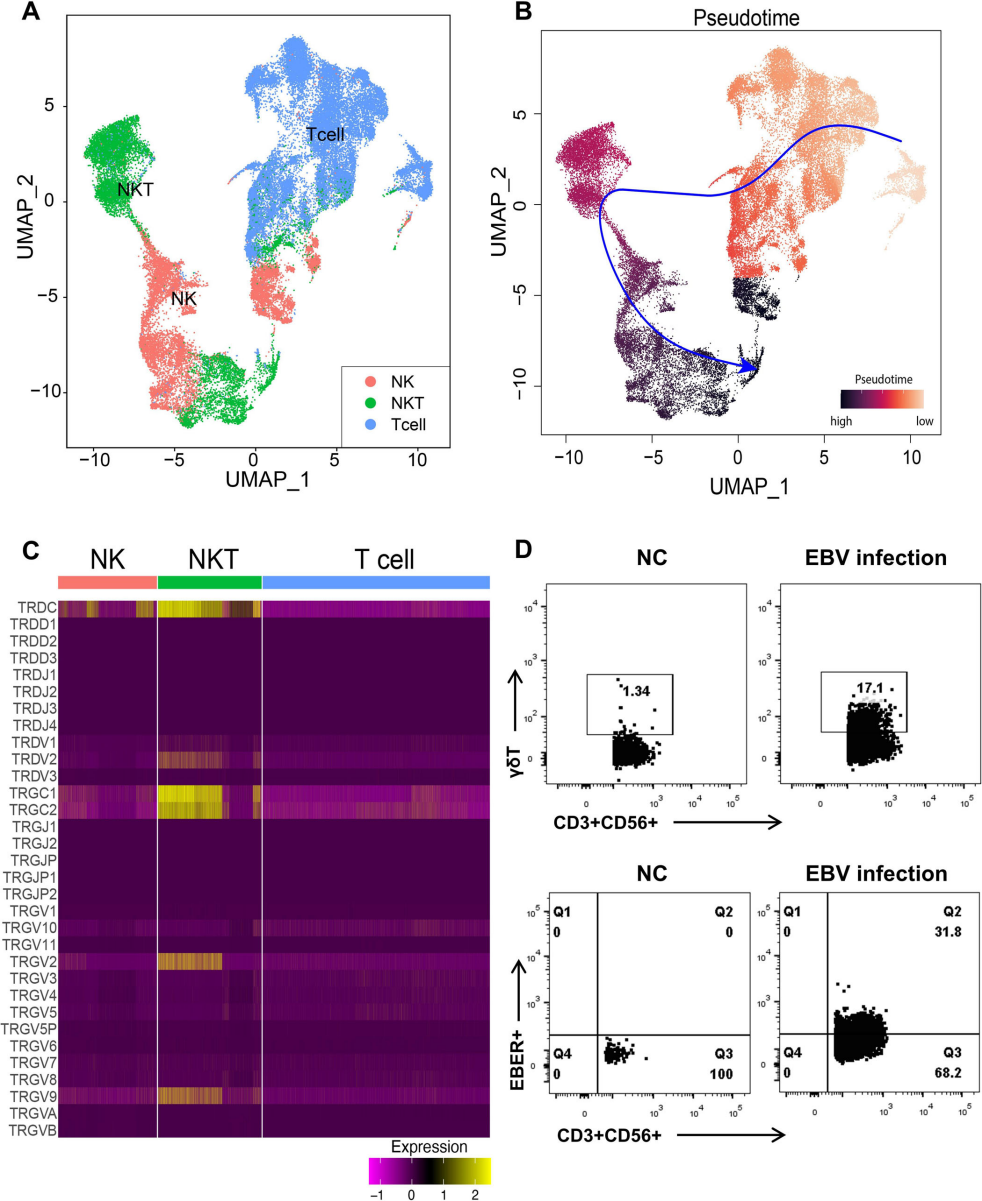
Pseudotime trajectory analysis traces the possible origin of NKT cells to T cells. (A) Re‐cluster the clusters into T, NKT, and NK cells. (B) Calculated pseudotime trajectory analysis based on differentially expressed genes in T, NKT, and NKT cells. The *arrow* is drawn according to the computed pseudotime coordinate, with lighter cells denoting earlier timepoints in the trajectory and dark cells denoting later timepoints. (C) Expression of γδ TCR genes among T, NKT, and NK cells. (D) EBV EBER RNA detected by flowRNA assay for in vitro EBV infected CD3 + CD56 + NKT cells (*lower panel*), and the expression of γδ TCR for EBV‐infected CD3 + CD56 + NKT cells (*upper panel*).

Whether NK/T lymphoma is derived from NK cells, which acquired CD3+ or derived from T cells that coexpressed CD56+ is unknown. The minor proportion of NKT cells in healthy donor's blood (average, 4%) [14] and the high percentage of NK and NKT cells in the subjects of the current study provided an opportunity to examine the functionality evolution between NK, NKT, and T cells. To testify this, the scRNA‐seq data for T, NK, and NKT cells of the current study were extracted and reclustered for pseudotime inferring (Figure 7A). The results of the current study showed that T cells were the starting point of all cells, which can be further differentiated into NKT cells (Figure 7B), along with the expression of CD56 (Supporting Information S1: Figure S4), indicating that NKT cells probably originated from T cells. Interestingly, NKT cells were found to be positive for γδ TCR gene expressions (Figure 7C), suggesting that a part of the NKT cells is γδ T phenotype. To confirm this, in vitro EBV infection experiments were performed and γδ chain expression was found to be higher in EBV‐infected CD3 + CD56 + T cells than in donor controls, indicating that EBV infection elevated the percentage of γδ T cells in vitro (Figure 7D).

## 4 Discussion

As some cases of EBV‐HLH patients experience a rapid relapse with mortality outcome after a short time of remission. The key factors drive this prognosis is still unknown. Thus, our study compared the surviving and deceased patients to explore the mechanism that driving the deterioration of EBV‐HLH. HLH was partly defined by lower NK functionality with decreased number of NK cells [15]. However, no threshold was defined for not only the absolute number but also the functionality of NK cells. In this study, a NK cell degranulation assay was conducted by measuring the CD107a+ NK cell counts to distinguish between healthy individuals and HLH patients. We found that all HLH patients had deficient NK cell degranulation ability at acute phase, which resolved upon recovery. In contrast, fatal cases showed a higher ability of degranulation and efficient cytotoxic function of NK cells at the deterioration phase, which agrees with the findings of Carvelli et al., who suggested that NK cell degranulation and cytotoxic functions are normal in patients with HLH [11]. Thus, the results of this study demonstrate that NK cell dysfunction was not the reason why HLH deteriorated rapidly.

Additionally, we showed that fatal cases had considerably effective EBV‐specific T cells when they deteriorated; therefore, the possibility that the lack of effective EBV‐specific T cells inhibited HLH progression was excluded. Meanwhile, one of the fatal cases in this study was found to exhibit a similar level of cytokines at the deterioration phase as the patient who recovered. Thus, we suggest that other factors may exist that drive HLH into the deterioration phase, except for the host cell immunity and cytokine storm. A new study showed that genetic defects, especially IFIH1 and/or DDX3X aberrations in the RLR pathway, were associated with unfavorable prognosis of EBV‐HLH [16]. While, we highlighted that high EBV loads were found in NK and/or NKT cells during the deterioration phase of the fatal cases with big amount of NK and NKT cells. These were supposed to drive the fatal outcome of patients.

However, the mechanism for the infection of EBV in NK or NKT cells which lack CD21 is not fully understood. NK cells were reported to acquire CD21 through synaptic transfer, which supports the binding of EBV to NK cells [17]. Others supposed that EBV may infect immature T cells or common progenitor cells, which can differentiate into T and NK cells through CD21 [18, 19]. While, we found that no CD21 expression was expressed in NK and NKT cells (Supporting Information S1: Figure S5A) and the expression of HLA‐DRA and HLA‐DRB1 genes was significantly higher in EBV‐infected NK and NKT cells than in noninfected NK and NKT cells (Supporting Information S1: Figure S5B,C). Thus, we suspect that EBV may use HLA‐DR as a receptor to enter NK and NKT cells in patients with HLH, which should be further investigated.

Furthermore, we found that the high load of EBV infection were accompany with higher level of NK and T cell cytotoxicity. Thus, infected NK and NKT cells are supposed to escape from or surpass host NK and T cell surveillance. Studies showed that EBV‐infected T cells present a monoclonal T cell clone expansion, which is different from EBV‐negative T cells that proliferate in high diversity [10, 20] suggesting that certain T cell clones were changed or transformed by EBV infection and then escaped the recognition from NK and EBV‐specific T cells under unknown circumstances. Besides, we noticed that the CNVs were highly presented in infected NK and NKT cells at the deterioration phase, and EBV‐infected cells had significantly higher enrichment in canonical cancer pathways, further indicating that EBV infection promoted the oncogenicity of NK and NKT cells, possibly contribute to the immune evasion of “self‐NK or NKT” cells.

To determine how EBV transformed NK/NKT cells in the early phase, the expression of the LMP1 gene in different subsets was investigated and the LMP1 gene was found to be dominantly present in infected NK and NKT cells at the deterioration phase, with the upregulation of EBV carcinogenesis‐related genes, such as *TRAF2* and *JAK3*, and the activation of the nuclear factor kappa B (NF‐κB) pathway. The results of the current study are consistent with those of a previous report that the viral protein LMP1 plays an important role in transforming infected B cells [21, 22, 23, 24]. However, the EBV carcinogenesis‐related gene *TRAAD* was downregulated in EBV‐infected NK and NKT cells compared with that in the noninfected group, as well as the *Bcl*2 gene; this is consistent with the phenomenon that *Bcl2* induction by LMP1 may be exclusive to B cells, which has no effect with the transfection of LMP1 into non‐B cells [25]. Thus, our study suggested that LMP1 transforms NK and NKT cells by increasing the expression of *TRAF2* with NF‐κB pathway activation, which need further investigation.

Our study has several limitations. The sample size for scRNA‐seq was relatively small. This limited sample size may restrict the generalizability of the findings. Besides, the patients were all from the First Affiliated Hospital of Guangzhou Medical University, resulting in a limited geographical and population source of the samples. This may limit the applicability of the research results in other regions or populations, and prevent their wide generalization. Therefore, larger multi‐center studies should be further conducted to provide more accurate and comprehensive insights.

In conclusion, the findings of this study indicate that despite the activation of NK and T cells, the clearance of infected NK/NKT cells in EBV‐HLH is not sufficient. EBV infection promotes the tumorigenesis of infected cells, leading to the rapid deterioration of HLH patients. These results provide valuable insights into the treatment of EBV‐related HLH and open up new avenues for future research in this area.

## Author Contributions

Z.W. designed and supervised the study. Z.W. and T.C. interpret data and drafted the manuscript. Y.W. performed scRNA‐seq analysis. T.C., M.H., Z.L., X.S., W.L., Q.L., and K.L. performed the experiment. R.Z. and C.W. recruited and treated the patients and contributed to the design of the study. All authors read and approved the final manuscript.

## Conflicts of Interest

The authors declare no conflicts of interest.

## Supporting information

Supplementary_Data‐tracking_version.

## Data Availability

The data that support the findings of this study are available on request from the corresponding author. The data are not publicly available due to privacy or ethical restrictions.
