# Supplementary material for: Oncogenic Potential of Epstein‐Barr Virus in NK and NKT Cells Contribute to the Rapid Deterioration of Hemophagocytic Lymphohistiocytosis
Source: J Med Virol. 2025 Jul 2;97(7):e70481. doi: 10.1002/jmv.70481 (PMC12216793; doi:10.1002/jmv.70481)

**Supplementary data**

**Materials and methods**

***NK cell cytotoxicity assay***

To directly assess NK cell cytotoxicity, a flow cytometry assay based on the GFP-K562 cells was performed. GFP-K562 cell line was generated by retrovirus transduction with K562 cells and the GFP-positive K562 cells were sorted using the FACSAria III instrument (BD Bioscience). GFP-K562 target cells were seeded in a V-bottomed well plate. PBMCs were added at effector/target (E: T) ratios of 20:1 and 5:1. Cells were incubated at 37^o^C for 3h in a humidified atmosphere of 5% CO_2_. Before analysis, annexin V-PE and 7-aminoactinomycin D (7-AAD, BD Pharmingen™) were added to stain dead cells. Then, cells were analyzed by the FACSAria III instrument (BD Bioscience). Only GFP-K562 cells without PBMCs were used as a control to determine spontaneous dead cells. Dead target cells were identified as annexin V positive within FITC positive cells.

***Infection of PBMCs with EBV in vitro***

To identify the EBV infection of PBMCs, in vitro infection assay was performed. PBMCs (3 x 106) were inoculated with EBV supernatant (107.3copies/ml) containing IL-12 (10ng/ml), IL-18(100ng/ml) and IL-2(10U/ml) for 24h at 37°C and 5% CO2. Thereafter, transformation medium containing cyclosporin (200ng/ml), IL-12, IL-18, and IL-2 was added to the cells which were further incubated. The EBV infection was analyzed by FowRNA assay at 18 days post inoculation. Meanwhile, the γδ chain expression of EBV infected cells was determined by Flow cytometry assay. Cells were stained with LIVE/DEAD Aqua Violet 510 for 15 min and surface-stained with anti-CD3-FITC (BioLegend, clone UCHT1), anti-CD4-APC-H7 (BD Pharmingen™, clone RPA-T4), anti-CD8-PerCPCy5.5 (BD Bioscience, clone RPA-T8), anti-γδ TCR-BV421 (BioLegend, clone B1) for 30 min at 4°C. Cells were acquired using the FACSAria III instrument (BD Biosciences) and analyzed using FlowJo, version 10.0 (Treestar).

**Supplementary Figure Legends**

**Supplementary Figure 1. Direct NK cell cytotoxicity of two deceased patients at the deterioration phase.** Natural cytotoxicity is represented by the different E:T ratios of P1 and P2.

**Supplementary Figure 2. Comparison of the functionality and cytokines between EBV infected and noninfected cells through high-throughput single-cell RNA-seq.** Expression of selected genes between EBV infected and noninfected NKT-1, NK-3, NK-7, and NKT-11 clusters.

**Supplementary Figure 3. Single-cell RNA-seq identifies oncogenesis of EBV-infected NK and NKT cells of EBV-HLH patients.** (A) Comparison of the enrichment of ten canonical pathways, including cell cycle, Hippo signaling, Myc signaling, Notch signaling, oxidative stress response/Nrf2, PI-3-Kinase signaling, receptor-tyrosine kinase (RTK)/RAS/MAP-Kinase signaling, TGFβ signaling, p53, and β-catenin/Wnt signaling, between EBV-infected and non-EBV-infected cells of P1 and P2. (B) Expression of selected genes representing proto-oncogenes, proliferation and EBV-viral carcinogenesis for EBV infected versus non-EBV clusters of three EBV-HLH patients. ns, not significant; *P < 0.05.

**Supplementary Figure 4. Pseudotime trajectory analysis traces the differentiation of CD56 positive cells.** Calculated pseudotime trajectory analysis based on CD56 expression in T, NKT, and NKT cells. The *arrow* is drawn according to the computed pseudotime coordinate, with lighter cells denoting earlier timepoints in the trajectory and dark cells denoting later timepoints.

**Supplementary Figure 5. Expression of CR2 (CD21) and HLA-DR in EBV-infected and EBV-noninfected cells by scRNA-seq.** (A) Expression of CD21 gene (CR2) in EBV infected and non-EBV infected NKT-1, NK-3, NK-7, and NKT-11 clusters. (B) Comparison of HLA-DRA gene expression between EBV infected and non-EBV infected NKT-1, NK-3, NK-7, and NKT-11 clusters. (C) Expression of HLA-DRB1 gene in EBV infected and non-EBV infected NKT-1, NK-3, NK-7, and NKT-11 clusters. ns, not significant; *P < 0.05.

**Figure S1**

**

**

**Figure S2**

**
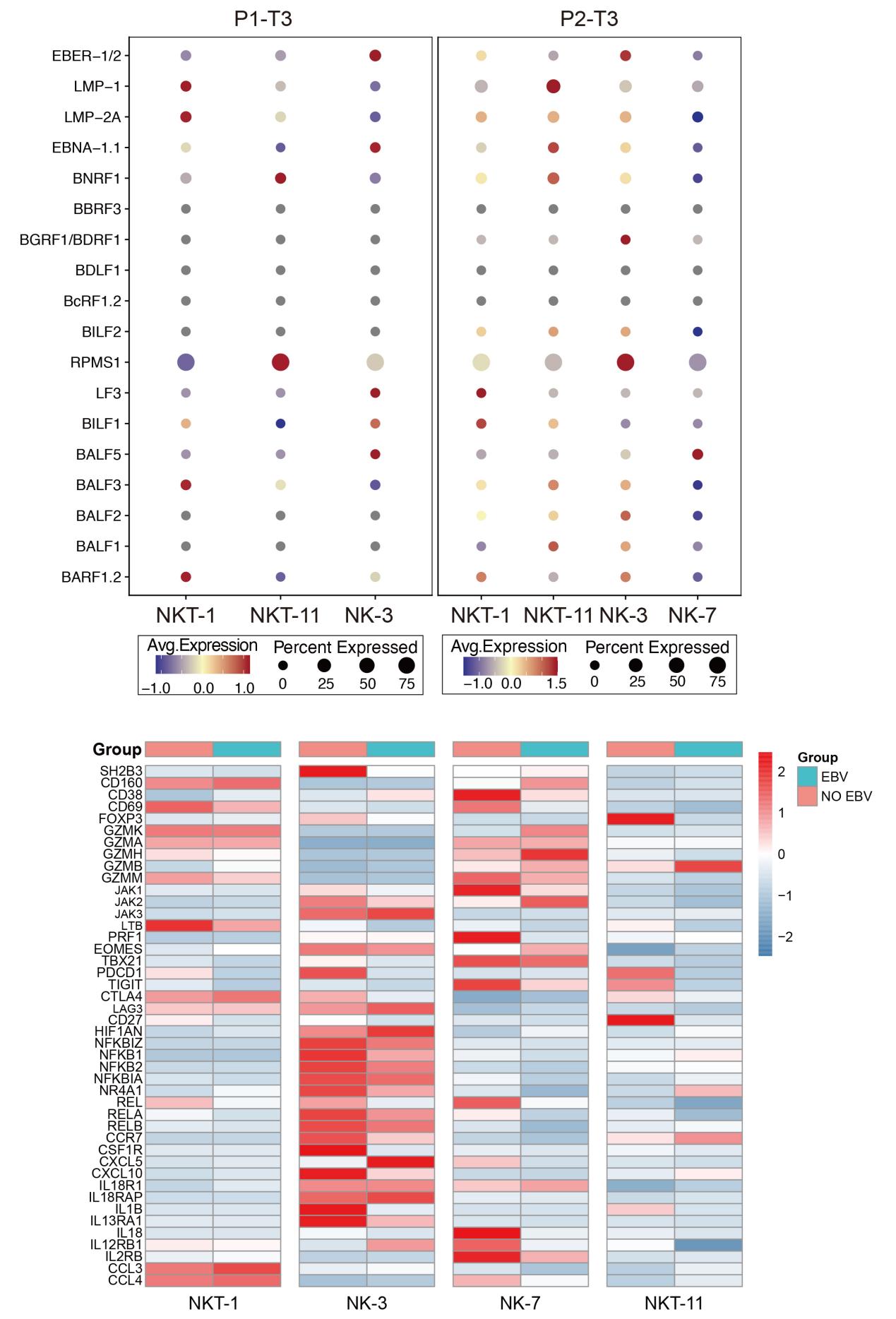
**

**Figure S3**

**
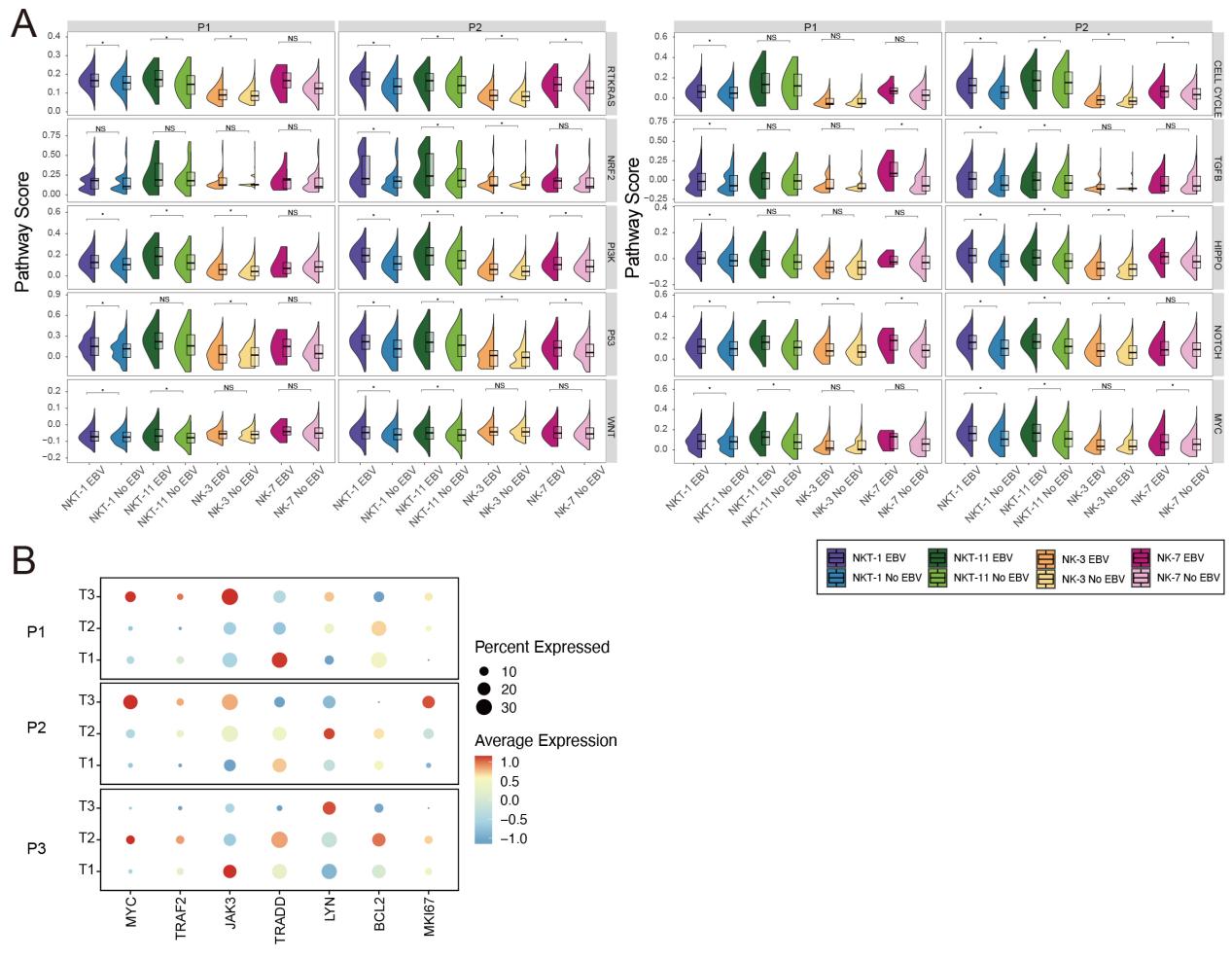
**

**Figure S4**

**
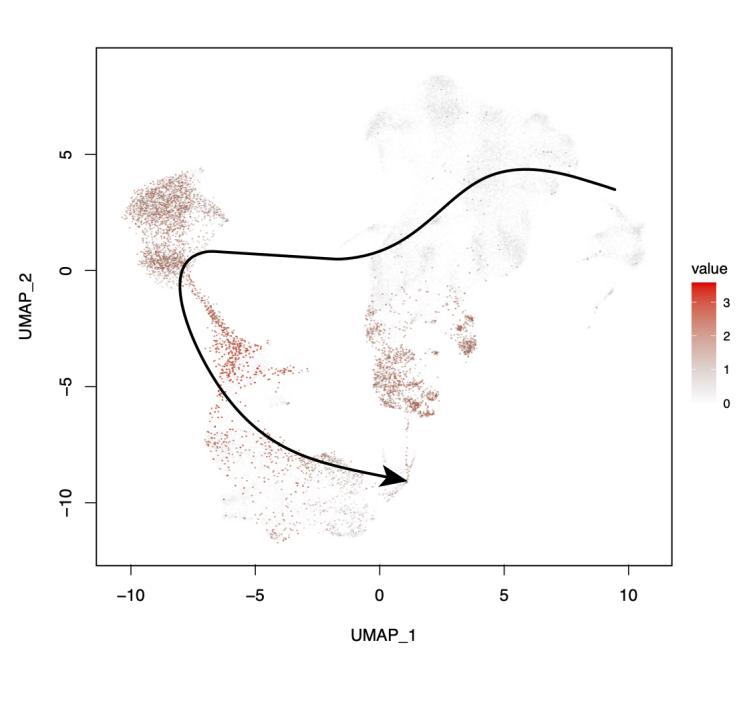
**

**Figure S5**


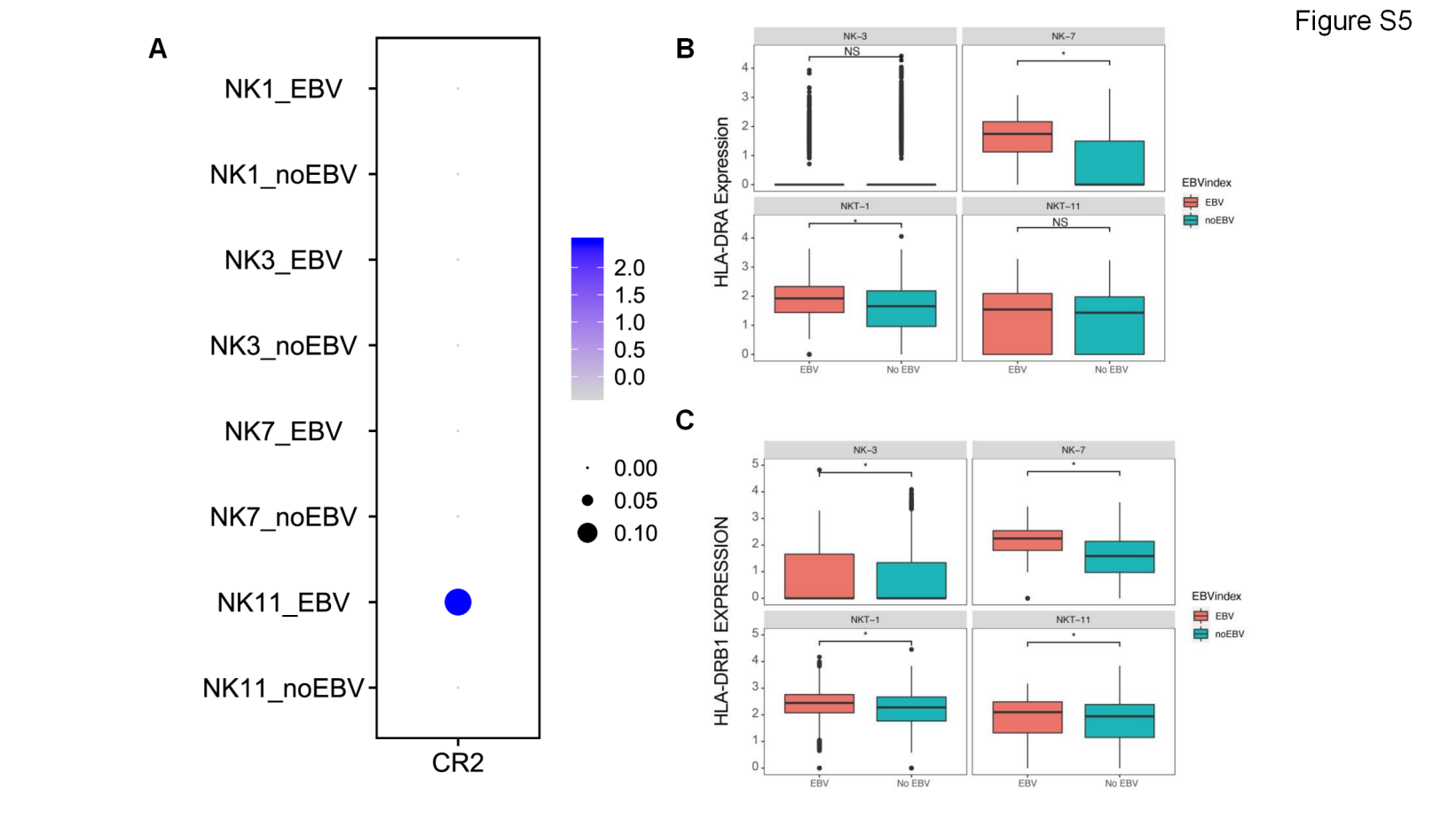

Supplement: Supplementary file 1 — Supplementary_Data‐tracking_version. [file JMV-97-e70481-s001.docx]
